# Supplementary material for: LSD1 inhibition circumvents glucocorticoid-induced muscle wasting of male mice
Source: Nat Commun. 2024 Apr 26;15:3563. doi: 10.1038/s41467-024-47846-9 (PMC11053113; doi:10.1038/s41467-024-47846-9)
Supplement: Supplementary file 1 — Supplementary Information [file 41467_2024_47846_MOESM1_ESM.pdf]

Supplementary Information

LSD1 inhibition circumvents glucocorticoid-induced muscle wasting

Qingshuang Cai, Rajesh Sahu, Vanessa Ueberschlag-Pitiot, Sirine Souali-Crespo, Céline Charvet, Ilyes Silem, Félicie Cottard, Tao Ye, Fatima Taleb, Eric Metzger, Roland Schuele, Isabelle M.L. Billas, Gilles Laverny, Daniel Metzger, Delphine Duteil

Supplementary figures

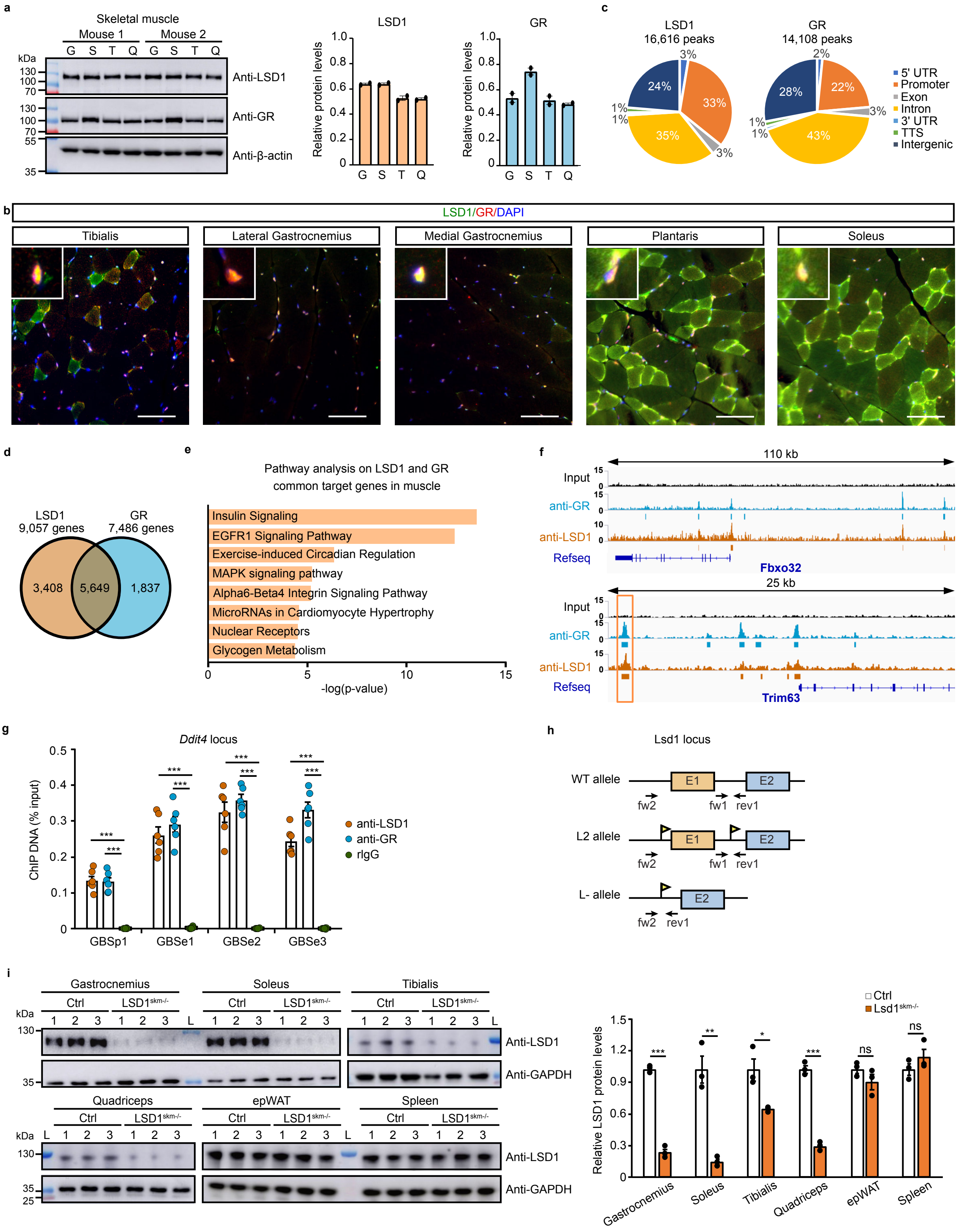

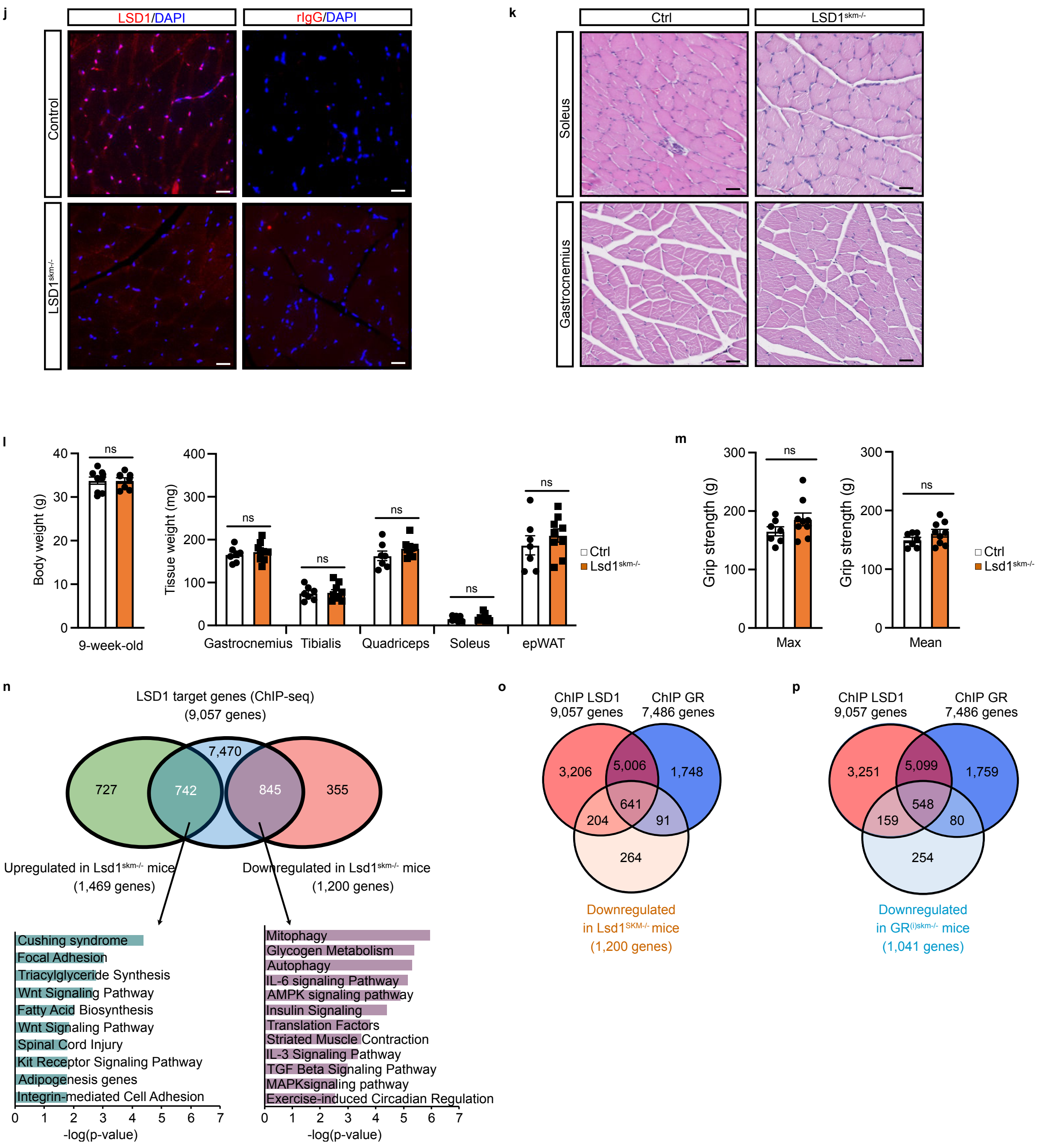

Supplementary Fig. 1. Control of gene expression by GR and LSD1 at physiological glucocorticoid levels.
a. Representative western blot analysis (left) and corresponding quantification (right) of LSD1 and GR protein levels in gastrocnemius (G), soleus (S), tibialis (T) and quadriceps (Q) muscles of 9-week-old wild type mice. β-actin was used as a loading control. **b.** Representative immunofluorescent detection of LSD1 (green) and GR (red) in tibialis, lateral and medial gastrocnemius, plantaris, soleus muscles of 9-week-old wild-type mice. Nuclei were stained with DAPI. Slow-twitch fibers are visible in green due to auto-fluorescence. A zoomed-in view of the confocal observation is shown on the right panel. Scale bar, 100 µm. N=3. **c.** Pie chart depicting the genomic location of LSD1 (left) and GR (right) binding in mouse skeletal muscles. **d.** Overlap between genes bound by LSD1 or GR in skeletal muscles. **e.** Pathway analysis on LSD1 and GR common target genes in skeletal muscles. **f.** Localization of GR and LSD1 at the *Fbxo32* and *Trim63* loci. The region of *Trim63* that will be hereafter considered for ChIP-qPCR analyses is boxed. **g.** ChIP-qPCR analysis at the indicated locations of the *Ddit4* locus with anti-LSD1 and anti-GR antibodies, or a rabbit IgG in skeletal muscle of wild-type mice. N=6. Mean ± SEM. Two-way ANOVA with Tukey correction. ***, p<0.001. **h.** Schematic representation of wild type (WT), floxed (L2) and Cre-mediated exon 1 deleted (L-) *Lsd1* alleles. LoxP sites are shown by arrowheads. Primers used for allele characterization are depicted by arrows and sequences are in Supplementary Table 1. **i.** Representative western

blot analysis (left) and corresponding quantification (right) of LSD1 protein levels in gastrocnemius, soleus, tibialis and quadriceps muscles, epididymal white adipose tissue (epWAT) and spleen of 9-week-old ctrl and LSD1$^{skm-/-}$ mice. GAPDH was used as a loading control. L: ladder. N=3 mice. Mean ± SEM. Two-tailed t-test. ns: non-significant; *, p<0.05; **, p<0.01; ***, p<0.001. **j.** Representative immunofluorescent detection of LSD1 or rabbit IgG (red) in gastrocnemius of 10-week-old control, LSD1$^{skm-/-}$ mice. Nuclei are stained with DAPI (blue). N=12. Scale bar, 50 µm. **k.** Representative H&E staining of soleus and gastrocnemius tissue of Ctrl and LSD1$^{skm-/-}$ mice. Scale bar, 50 µm. N=3. **l.** Body, gastrocnemius, tibialis, quadriceps and soleus muscles, and epWAT mass of 9-week-old Ctrl and LSD1$^{skm-/-}$ mice. N=7 Ctrl and 9 LSD1$^{skm-/-}$ mice. Mean ± SEM. Two-tailed t-test. ns ns: non-significant. **m.** Maximal (Max) and average (Mean) grip strength of 9-week-old Ctrl and LSD1$^{skm-/-}$ mice. N=7 Ctrl and 9 LSD1$^{skm-/-}$ mice. Mean ± SEM. Two-tailed t-test. ns: non-significant. **n.** Overlap between genes bound by LSD1 in skeletal muscle, and genes that are either up- or down-regulated in skeletal muscles of LSD1$^{skm-/-}$ mice, and corresponding pathway analysis of direct targets. **o-p.** Overlap between genes bound by GR and LSD1 in skeletal muscle, and down-regulated in skeletal muscles of LSD1$^{skm-/-}$ (o) or GR$^{(i)skm-/-}$ (p) mice. Source data are provided as a Source Data file.

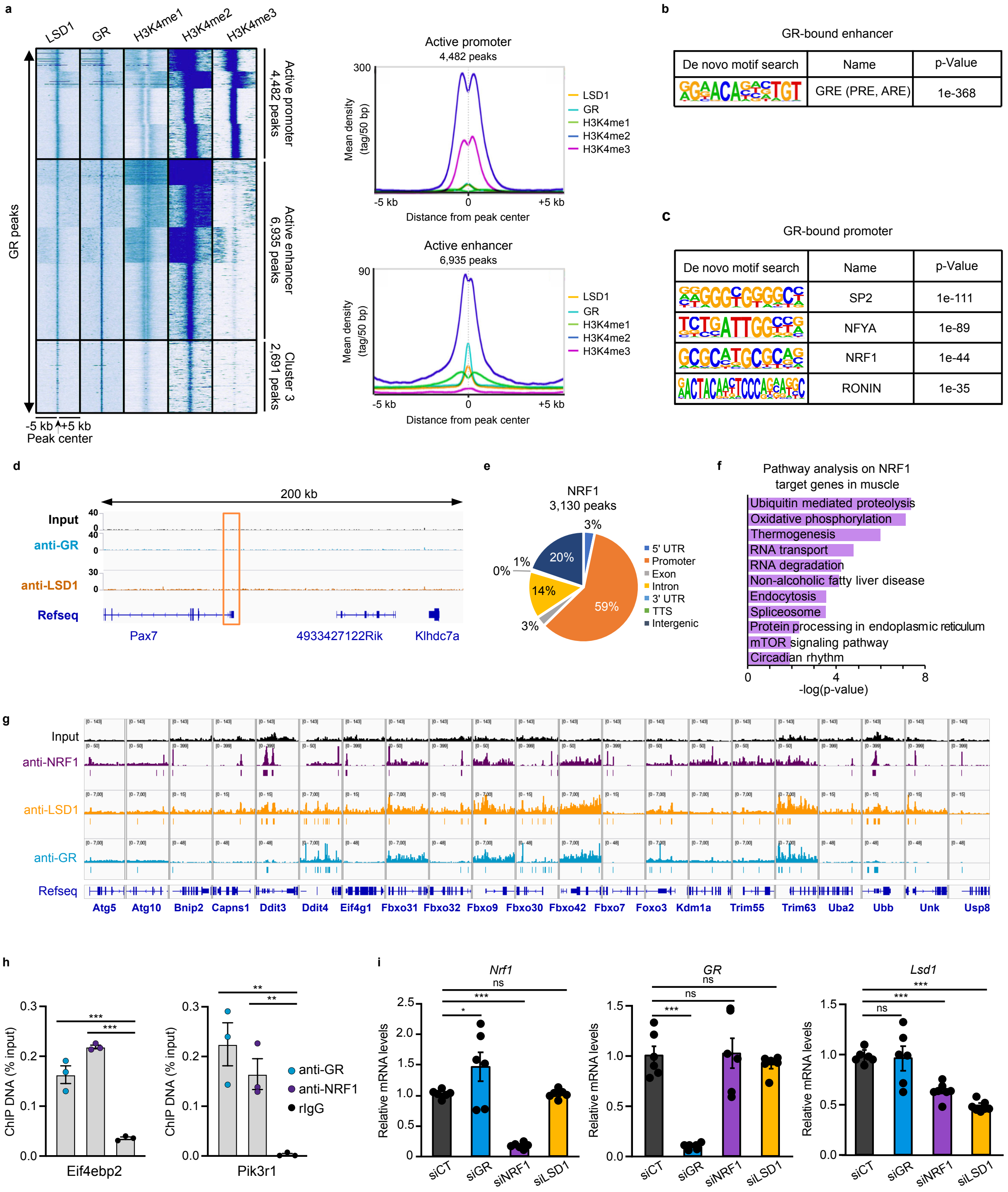

Supplementary Fig. 2. Analysis of LSD1, GR and NRF1 interactions and genomic distribution in mouse skeletal muscle

a. Tag density map of LSD1, GR, H3K4me1, H3K4me2 and H3K4me3 in mouse skeletal muscles, ± 5 kb from the GR peak center and corresponding average tag density profiles. **b-c.** HOMER *de novo* motif analysis of GR binding sites located at enhancer (b) and promoter regions (c). **d.** Localization of GR and LSD1 at the *Pax7* locus. The region considered for ChIP-qPCR analyses is boxed in orange. **e.** Pie chart depicting the genomic location of NRF1 binding in mouse skeletal muscles. **f.** Pathway analysis of NRF1 target genes in skeletal muscle of wild-type mice at 9 weeks. **g.** Localization of GR, LSD1 and NRF1 at the indicated gene loci. **h.** ChIP-qPCR analysis performed with anti-GR and anti-NRF1 antibodies or rabbit IgG at the indicated gene loci. One-way ANOVA with Tukey correction. **, p<0.001; ***, p<0.001. **i.** Relative *Nrf1, Gr* and *Lsd1* transcript levels determined in C2C12 myotubes transfected siRNA directed against *Gr*, *Nrf1* and *Lsd1* (siGR, siNrf1 or siLSD1, respectively), or with a

scramble siRNA (siCtrl). Mean ± SEM. One-way ANOVA with Tukey correction. ns: non-significant; *, p<0.05. ***, p<0.001. Source data are provided as a Source Data file.

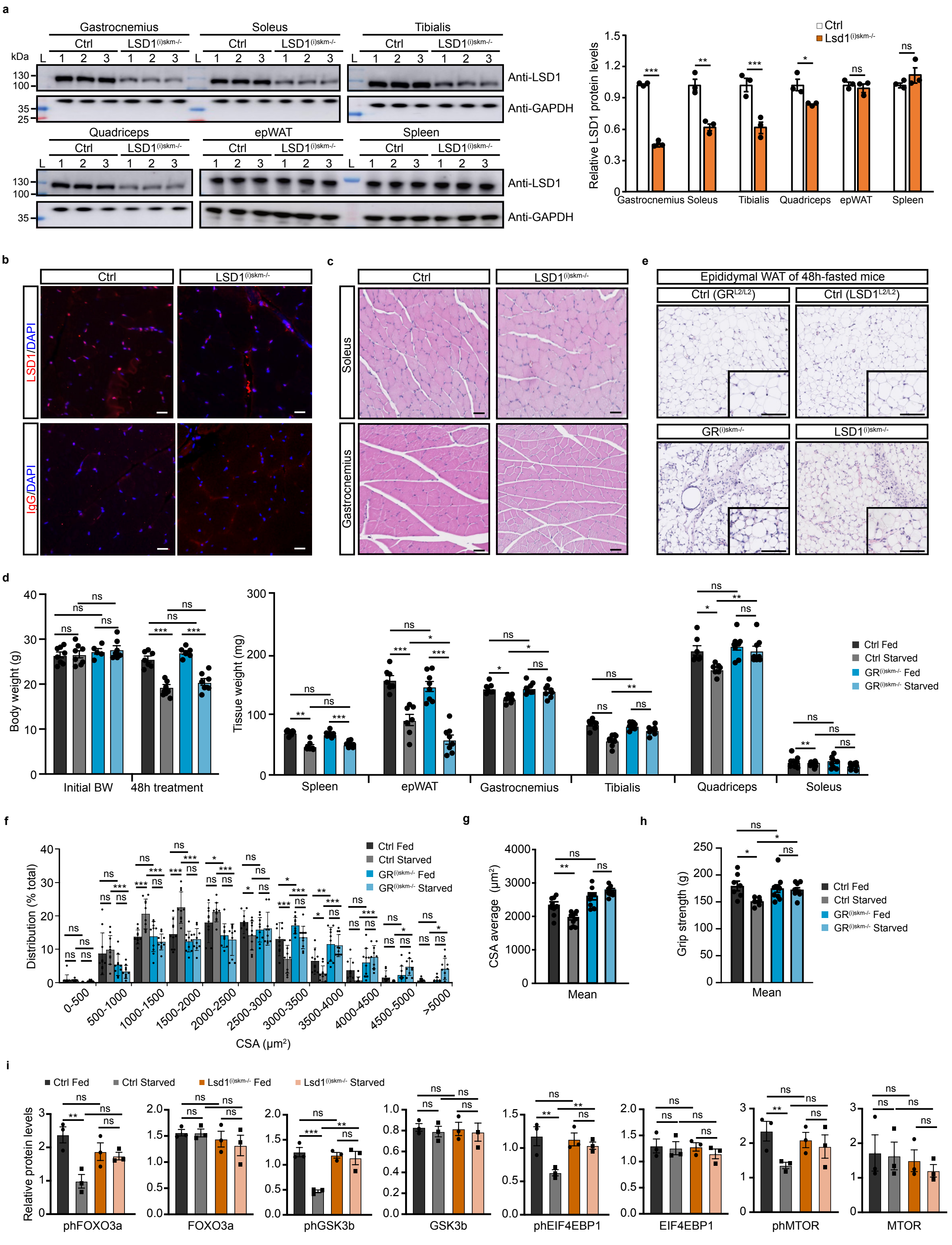

a
Gastrocnemius
Soleus
Tibialis
Ctrl
LSD1$^{(i)skm-/-}$
kDa
L 1 2 3 1 2 3
130
100
35
25
Anti-LSD1
Anti-GAPDH
Quadriceps
epWAT
Spleen
Relative LSD1 protein levels
Ctrl
Lsd1$^{(i)skm-/-}$
Gastrocnemius Soleus Tibialis Quadriceps epWAT Spleen
b
LSD1/DAPI
IgG/DAPI
c
Soleus
Gastrocnemius
e
Epididymal WAT of 48h-fasted mice
Ctrl (GR$^{L2/L2}$)
Ctrl (LSD1$^{L2/L2}$)
GR$^{(i)skm-/-}$
LSD1$^{(i)skm-/-}$
d
Body weight (g)
Initial BW
48h treatment
Tissue weight (mg)
Spleen
epWAT
Gastrocnemius
Tibialis
Quadriceps
Soleus
Ctrl Fed
Ctrl Starved
GR$^{(i)skm-/-}$ Fed
GR$^{(i)skm-/-}$ Starved
f
Distribution (% total)
0-500
500-1000
1000-1500
1500-2000
2000-2500
2500-3000
3000-3500
3500-4000
4000-4500
4500-5000
>5000
CSA (μm²)
g
CSA average (μm²)
Mean
h
Grip strength (g)
Mean
i
Ctrl Fed
Ctrl Starved
Lsd1$^{(i)skm-/-}$ Fed
Lsd1$^{(i)skm-/-}$ Starved
Relative protein levels
phFOXO3a
FOXO3a
phGSK3b
GSK3b
phEIF4EBP1
EIF4EBP1
phMTOR
MTOR

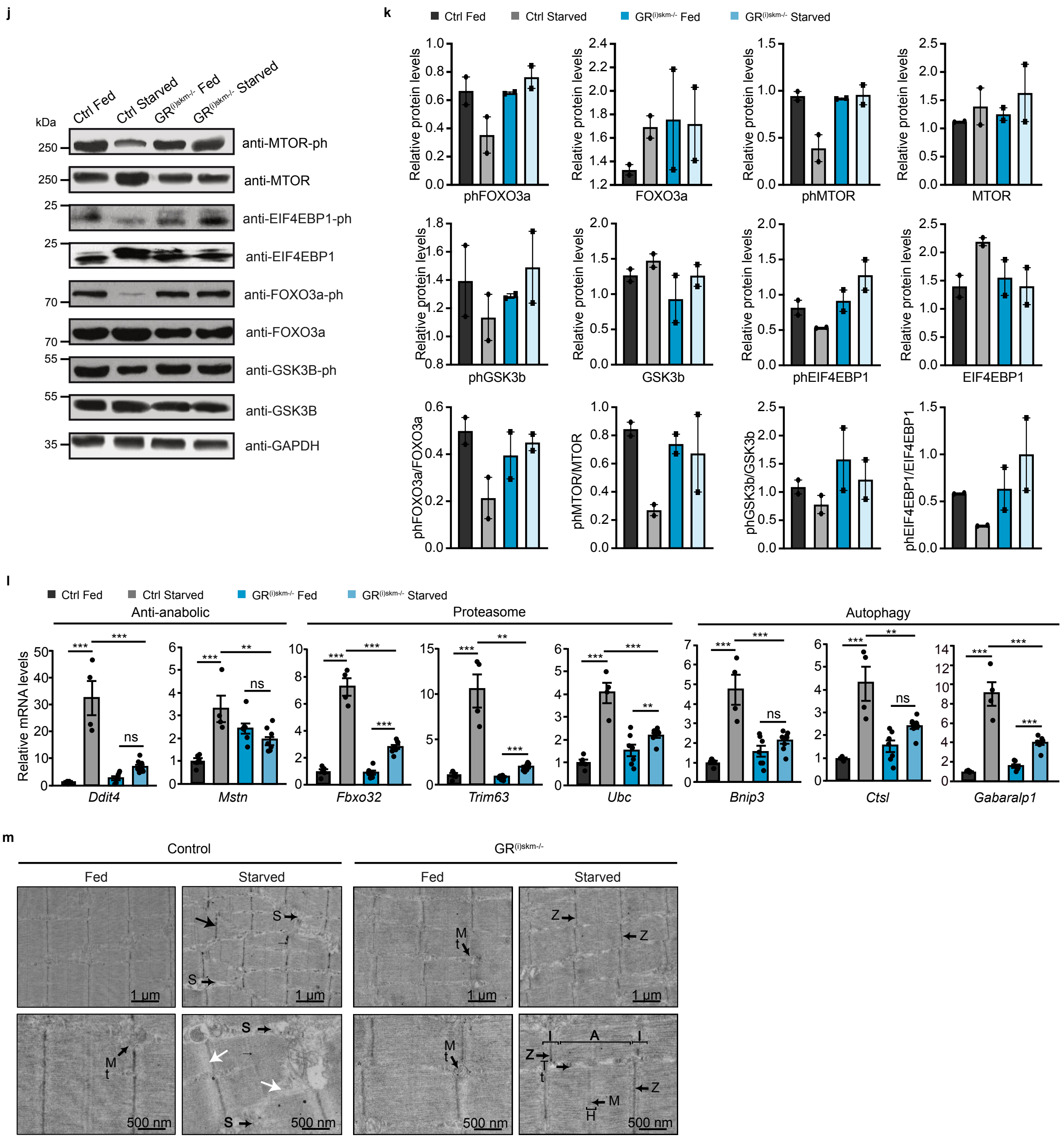

Supplementary Fig. 3. Role of GR and LSD1 in starvation-induced muscle wasting.

a. Representative western blot analysis (left) and corresponding quantification (right) of LSD1 protein levels in gastrocnemius, soleus, tibialis and quadriceps muscles, epWAT and spleen of 12-week-old ctrl and LSD1$^{(i)skm-/-}$ mice. GAPDH was used as a loading control. L: Ladder. N=3 mice. Mean ± SEM. Two-way ANOVA with Tukey correction. ns: non-significant; *, p<0.05; **, p<0.01; ***, p<0.001. **b.** Representative immunofluorescent detection of LSD1 or rabbit IgG (red) in gastrocnemius of 10-week-old Ctrl and LSD1$^{(i)skm-/-}$ mice. Nuclei are stained with DAPI (blue). N=12. Scale bar, 50 µm. **c.** Representative H&E staining of soleus and gastrocnemius muscles of Ctrl and LSD1$^{(i)skm-/-}$ mice. Scale bar, 50 µm. N=3. **d.** Body, spleen, epWAT gastrocnemius, tibialis, quadriceps and soleus muscle mass of 12-week-old Ctrl and GR$^{(i)skm-/-}$ mice fed or starved for 48 h. N=7 Ctrl Fed, 7 Ctrl Starved, 8 GR$^{(i)skm-/-}$ Fed and 8 GR$^{(i)skm-/-}$ Starved mice. Mean ± SEM. Two-way ANOVA. ns: non-significant; *, p<0.05; **, p<0.01; ***, p<0.001. **e.** Representative H&E staining of epWAT of 12-week-old Ctrl, GR$^{(i)skm-/-}$ and LSD1$^{(i)skm-/-}$ mice starved for 48 h. Scale bar, 50 µm. N=3. **f-h.** Distribution of fiber CSA (f) and average CSA (g) in gastrocnemius, and average (mean) grip strength (h) of 12-week-old Ctrl and GR$^{(i)skm-/-}$ mice fed or starved for 48h. N=9. Mean ± SEM. Two-way ANOVA with Tukey correction. ns: non-significant; *, p<0.05; **, p<0.01; ***, p<0.001. **i.** Relative levels of the indicated proteins in quadriceps muscle of 12-week-old Ctrl and LSD1$^{(i)skm-/-}$ mice fed or starved for 48 h. N = 3 mice. Mean ± SEM. Two-way ANOVA with Tukey correction. ns: non-significant;

p < 0.01; ***p < 0.001. **j-k. Representative western blot analysis (j) and relative quantification (k) of the indicated proteins in quadriceps muscle of 12-week-old Ctrl and $GR^{(i)skm-/-}$ mice fed or starved for 48 h. GAPDH was used as a loading control. **l.** Relative transcript levels of the indicated genes in gastrocnemius muscle of 12-week-old Ctrl and $GR^{(i)skm-/-}$ mice fed or starved for 48 h. N=5 Ctrl fed, 4 Ctrl starved, 6 $GR^{(i)skm-/-}$ fed and 8 $GR^{(i)skm-/-}$ starved mice. Mean ± SEM. Two-way ANOVA with Tukey correction. ns: non-significant; **, p<0.01, ***, p<0.001. **m.** Ultrastructure analysis of gastrocnemius muscles of 12-week-old Ctrl and $GR^{(i)skm-/-}$ mice fed or starved for 48 h. A, A band; I, I band; H. H band; M, M line; Mt, mitochondria; Tt, T-tubule; S, sarcoplasm; Z, Z line. Black arrow indicates Z line disruption; white arrows indicate loss of myofilaments. Source data are provided as a Source Data file.

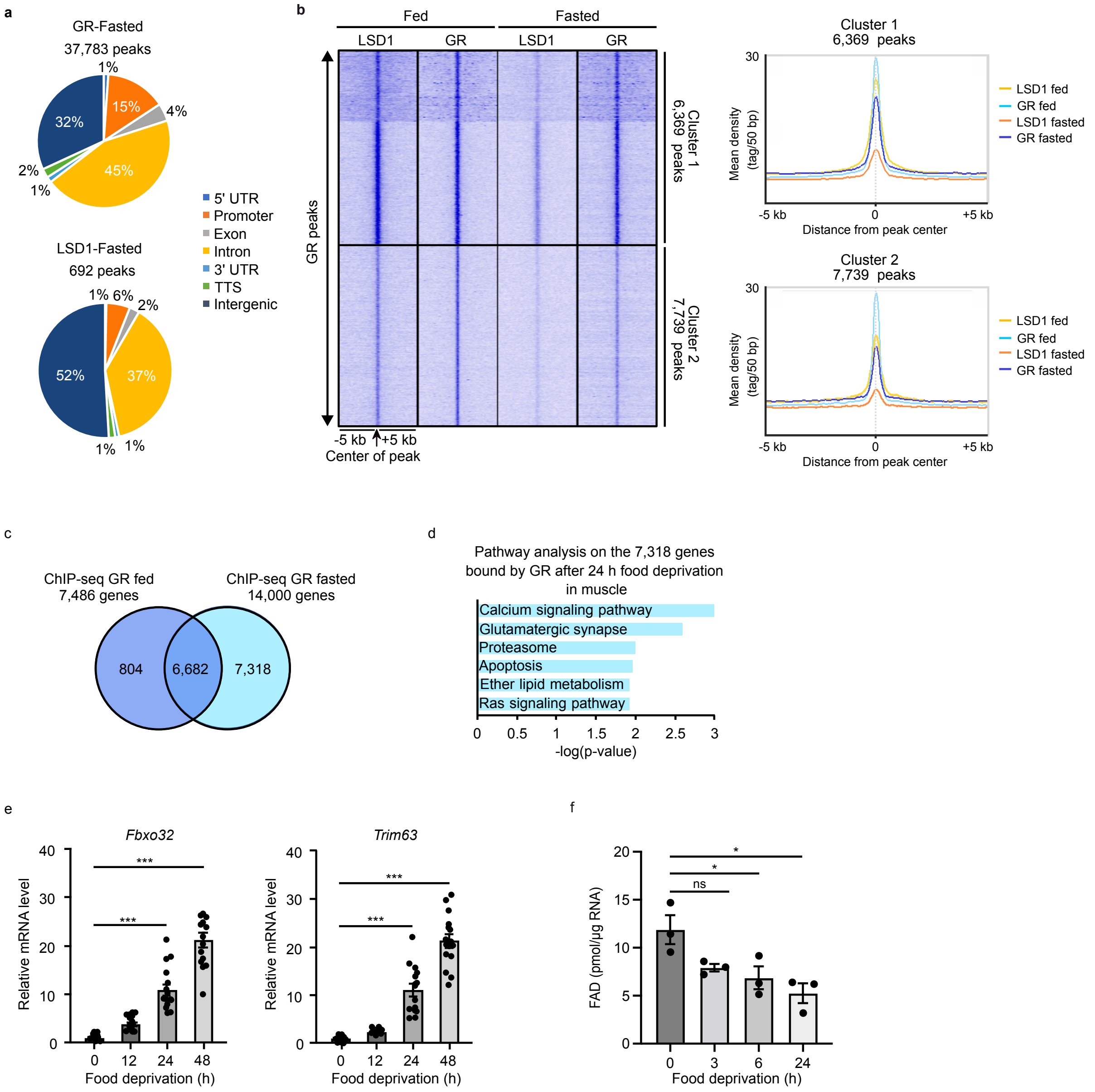

Supplementary Fig. 4. Analysis of LSD1 and GR binding dynamics in skeletal muscle during food deprivation. **a.** Pie chart depicting the genomic location of LSD1 and GR binding in skeletal muscles of wild-type mice fasted for 24 h. **b.** Tag density map of LSD1 and GR in skeletal muscles of mice fed or fasted for 24 h, ± 5 kb from the GR peak center and corresponding average tag density profiles. **c.** Overlap between genes bound by GR fed and fasted in skeletal muscle. **d.** Pathway analysis on the genes targeted by GR only in skeletal muscles 24 h after food deprivation. **e.** Relative transcript levels of *Fbxo32* and *Trim63* in gastrocnemius of wild-type mice fed or food deprived for 12, 24 or 48 h. N=13-15. Mean ± SEM. Two-way ANOVA with Tukey correction. ***, p<0.001. f. FAD levels in quadriceps muscles of wild-type mice after 0, 3, 6 and 24 h of food deprivation. N=3. Mean ± SEM. Two-way ANOVA with Tukey correction. ns: non-significant; *, p<0.05. Source data are provided as a Source Data file.

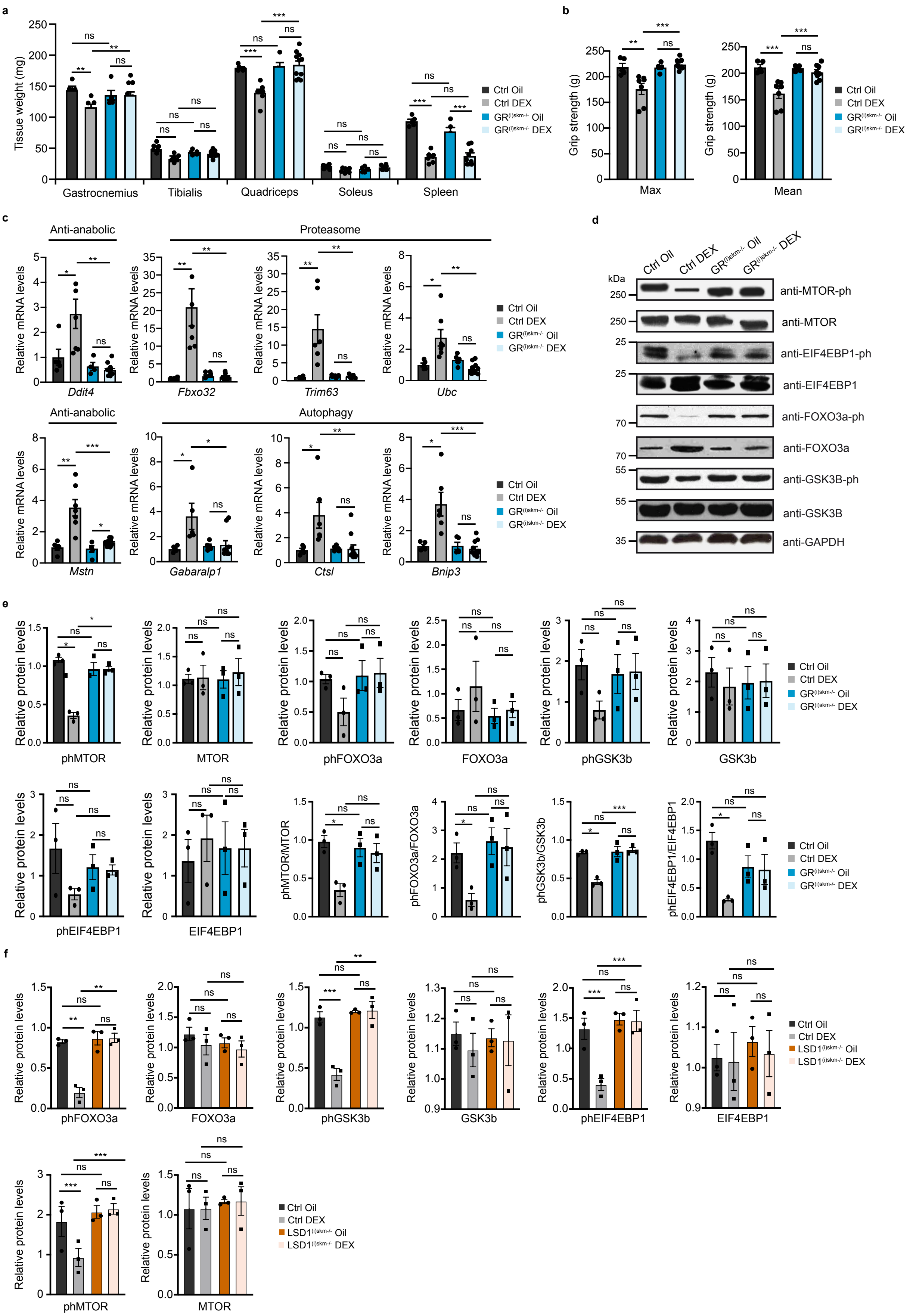

Supplementary Fig. 5. LSD1 and GR are required for dexamethasone-induced muscle wasting

a. Gastrocnemius, tibialis, quadriceps, soleus and spleen mass of Ctrl and GR$^{(i)skm-/-}$ mice treated with dexamethasone (DEX) or vehicle (Oil) for 72 h. N=5 Ctrl Oil, 6 Ctrl DEX, 5 GR$^{(i)skm-/-}$ Oil and 11 GR$^{(i)skm-/-}$ DEX mice.

Mean ± SEM. Two-way ANOVA with Tukey correction. ns: non-significant; **, p<0.01; ***, p<0.001. **b.** Maximal (Max) and average (Mean) grip strength of Ctrl and $GR^{(i)skm-/-}$ mice treated with DEX or Oil for 72 h. N=5 Ctrl Oil, 7 Ctrl DEX,5 $GR^{(i)skm-/-}$ Oil and 9 $GR^{(i)skm-/-}$ DEX mice. Mean ± SEM. One-way ANOVA with Tukey correction. ns: non-significant; **, p<0.01; ***, p<0.001. **c.** Relative transcript levels of the indicated genes in gastrocnemius of Ctrl and $GR^{(i)skm-/-}$ mice treated with DEX or Oil for 72 h. N=5 Ctrl Oil, 7 Ctrl DEX, 5 $GR^{(i)skm-/-}$ Oil and 8 $GR^{(i)skm-/-}$ DEX mice. Mean ± SEM. Two-way ANOVA with Tukey correction. ns: non-significant; *, p<0.05; **, p<0.01; ***, p<0.001. **d-e.** Representative western blot analysis (d) and corresponding quantification (e) of the indicated proteins in quadriceps muscle of 12-week-old Ctrl and $GR^{(i)skm-/-}$ mice treated with DEX or Oil for 72h. GAPDH was used as a loading control. n = 3 mice. Mean ± SEM. Two-way ANOVA with Tukey correction. ns: non-significant; *, p<0.05. **, p<0.01; ***, p<0.001. **f.** Relative levels of the indicated proteins in quadriceps muscle of 12-week-old Ctrl and $LSD1^{(i)skm-/-}$ mice treated with DEX or Oil for 72h. Source data are provided as a Source Data file.

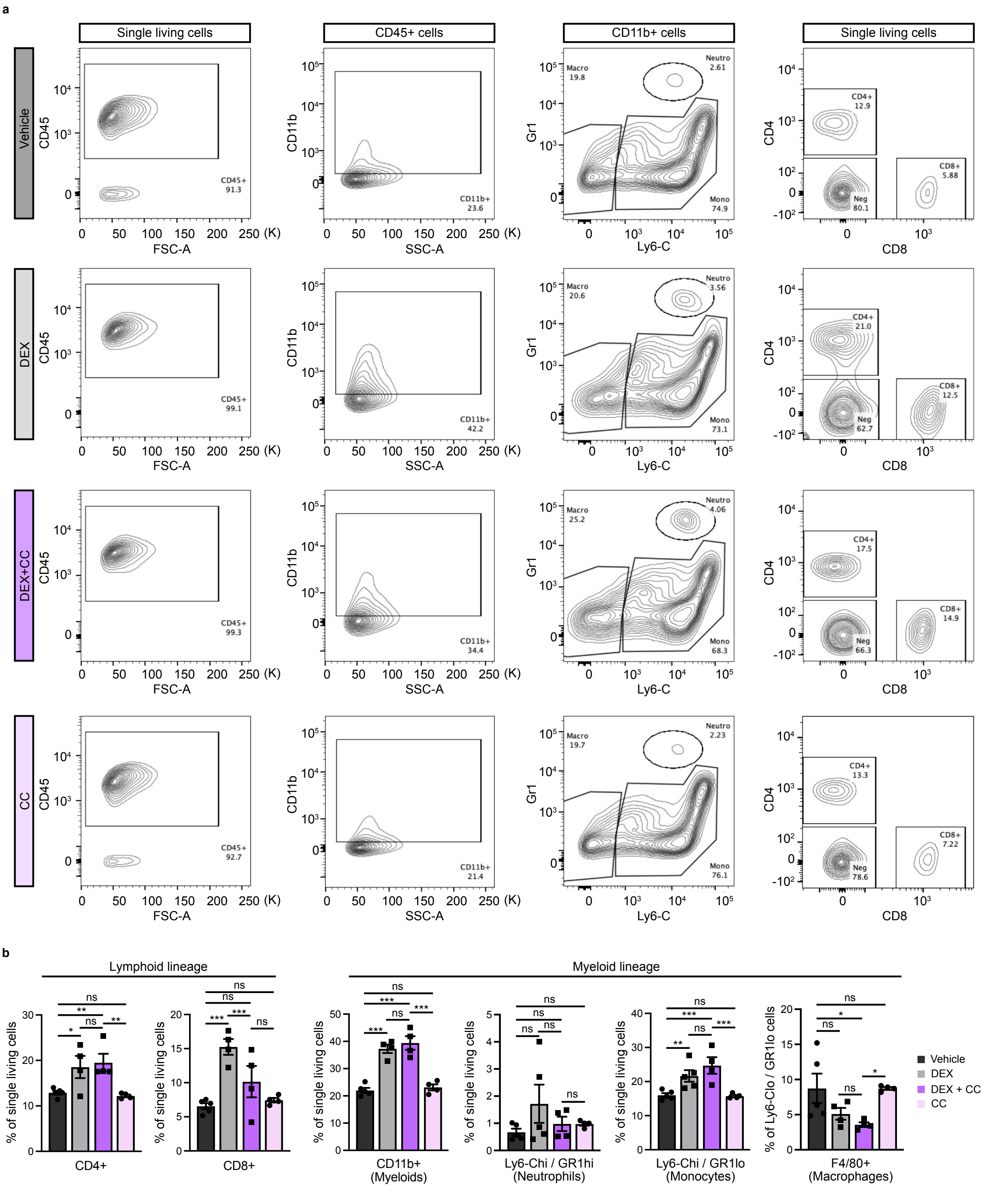

Supplementary Fig. 6. Effect of LSD1 inhibition on dexamethasone anti-inflammatory activities.

a-b. Flow cytometry analysis of the lymphoid and the myeloid lineages in spleen of wild-type mice treated with a vehicle, DEX, DEX with CC-90011 (DEX+CC) or CC-90011 (CC) for 72 h (a) and corresponding quantification (b). Ly6-Chi: high levels of Ly6-C, Ly6-Clo: low levels of Ly6-C, GR1lo: low levels of GR1, GR1hi: high levels of GR1. N=5 vehicle, 4 DEX, 4 DEX+CC, 4 CC mice. Mean ± SEM. One-way ANOVA with Tukey correction. ns: non-significant; *, p<0.05; **, p<0.01; ***, p<0.001. Source data are provided as a Source Data file.

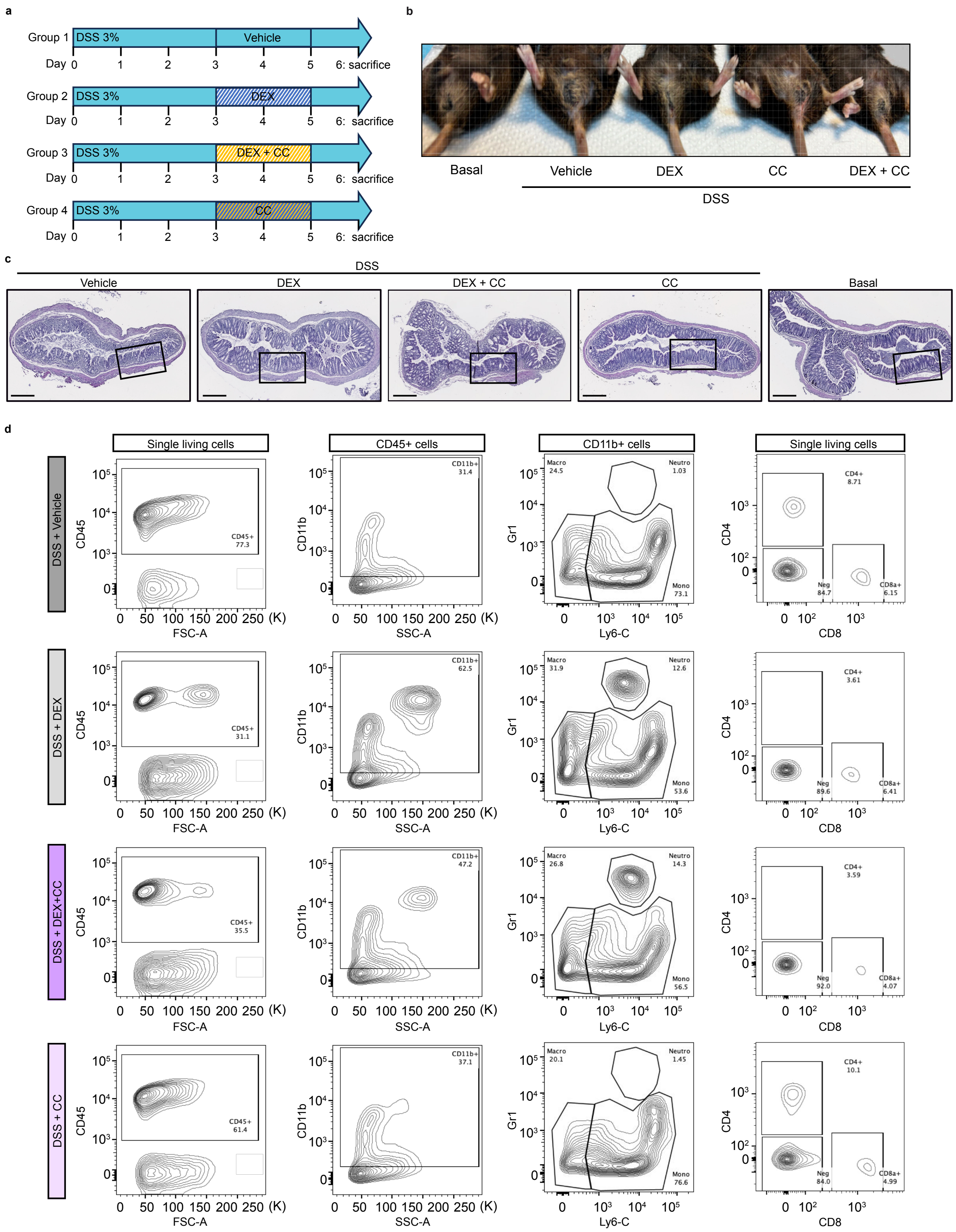

Supplementary Fig. 7. Effect of LSD1 inhibition on dexamethasone-induced amelioration of experimental colitis.

a. Flow chart illustrating study design. Mice were treated with 3% DSS in drinking water over a six-day period to induce colitis, DEX and/or CC-90011 were administrated from the 3rd to the 5th day of colitis. N=5 mice per condition. **b.** Representative colitis syndrome with diarrhea, and/or hematochezia in mice from indicated groups. **c.** Representative H&E staining of colons in mice from indicated groups. Scale bars, 200 µm. This experiment has been performed on 3 mice per group. **d.** Flow cytometry analysis of the lymphoid and the myeloid lineages in spleen of mice from indicated groups.

Supplementary Tables

Supplementary Table 1. List of the primers used for genotyping.

Name	Forward (5'-3')	Reverse primer (5'-3')
Cre and CreER^{T2} recombinase alleles	TTCCCGCAGAACCTGAAGATGTTCG	GGGTGTTATAAGCAATCCCCAGAAATGC
GR WT/L2 allele	AGATCATTTGCCTAGCAGGCATGAG	GTCAACACATGATCACCTTGCAGTC
GR L- allele	CCAGAGAACTAATTGGCTCTTGCAC	GTCAACACATGATCACCTTGCAGTC
Lsd1 WT/L2 allele (fw1-rev1)	CCTCAGTAGGCCTGGTTTGT	TTGGTTTTGGTTGACCCTTC
Lsd1 L- allele (fw2-rev1)	CCGTGGAAATTCGTGCACTC	GCAGGCGGTTTGAAATGTATTC

Supplementary Table 2. List of the primers used for RT-qPCR analyses.

Gene name	Forward (5’-3’)	Reverse primer (5’-3’)
m18S	TCGTCTTCGAAACTCCGACT	CGCGGTTCTATTTTGTTGGT
m36b4	AGATTCGGGATATGCTGTTGG	AAAGCCTGGAAGAAGGAGGTC
mAtg7	CCACTGAGGTTCACCATCCT	TCCGTTGAAGTCCTCTGCTT
mBecn1	GAGCCATTTATTGAAACTCGCCA	CCTCCCCGATCAGAGTGAA
mBnip3	TTCCACTAGCACCTTCTGATGA	GAACACCGCATTTACAGAACAA
mCtsl	GTGGACTGTTCTCACGCTCAAG	TCCGTCCTTCGCTTCATAGG
mDdit4	TGGTGCCCACCTTTCAGTTG	GTCAGGGACTGGCTGTAACC
mFbxo32	TCAGCCTCTGCATGATGTTC	TCAGCCTCTGCATGATGTTC
mGabaralp1	CATCGTGGAGAAGGCTCCTA	ATACAGCTGGCCCATGGTAG
mHprt	GTTGGATACAGGCCAGACTTTGTTG	GATTCAACTTGCGCTCATCTTAGGC
mMstn	GGCCATGATCTTGCTGTAAC	TTGGGTGCGATAATCCAGTC
mTrim63	TGAGGTGCCTACTTGCTCCT	GTGGACTTTTCCAGCTGCTC
mUbc	TCTTCGTGAAGACCCTGACC	CAGGTGCAGGGTTGACTCTT
mIL-6	TCTGCAAGAGACTTCCATCCA	AGTCTCCTCTCCGGACTTGT

Gene name	Forward (5’-3’)	Reverse primer (5’-3’)
hATG7	TCCCATGTGTTCAGTTCACCC	AACAGCCATTTTGCCACTAATC
hCTSL	GTGGACATCCCTAAGCAGGA	CACAATGGTTTCTCCGGTC
hDDIT4	TGTTTAGCTCCGCCAACTC	TTCTTGATGACTCGGAAGCC
hFBXO32	GACTTCTCAACTGCCATTC	TCGTCTCCATCCGATACAC
hGABARALP1	TGAGACCTGAGGACGCCTT	GGGCTTCCAACCACTCATTTC
hGADD45A	CGTTTTGCTGCGAGAACGAC	GAACCCATTGATCCATGTAG
hMSTN	GATGACGATTATCACGCTAC	GCACAAACACTGTTGTAGGA
hRPLP0	CGTCCTCGTGGAAGTGACAT	TAGTTGGACTTCCAGGTCGC
hTRIM63	CGTGGAGATGAAAACCGACC	GGCCCACTCTAGACTTGGTT

Supplementary Table 3. List of the primers used for ChIP-qPCR experiments.

Locus	Forward (5'-3')	Reverse primer (5'-3')
Ddit4_GBSp1	AGGCGCTAAGAATCGGAGTC	CACGCTGTCTTTGCTTCTGA
Ddit4_GBSe1	AAGAAGGCAGGTCTCCACTC	GTTACCCTGGGCTGAAGTCT
Ddit4_GBSe2	TAGAACACACCCAGCAGAGG	AGCCAGAGGTAGGAGAGACA
Ddit4_GBSe3	CTGTGGGTGGAAGGATGCT	ACTCCCTGTTTACACGGTGT
Eif4ebp2_GBSe2	ACAACCTTGACATCCACCCA	GGTGCATCTGGTGGAATGTG
Eif4ebp2_GBSp2	AGAGCAGGCGAGTTGAGAG	GTAGTCCTGAGGTAGCTGCG
Pax7	CCCCAGGCTAGAACCATTG	ATAGAGCTGCCTTGTTCCC
Pik3r1_GBSe3	TTCTTTCTCCCGTCTGTGCT	TGGGCAGTGGGAATAAAGGT
Murf1_5kb	GCTGAGTTCCTGGGACACTC	GGGTTCTGGGTCATGATGCA
Unrelated region (Eif4ebp2 intron)	CGGATTTGGAGTTCAGCCTG	CCCCTTCCTTGTTTGGTTGG

Supplementary Table 4. List of the antibodies used for spleen FACS analysis.

Antigen	Conjugation	Company	Cat. number	Dilution
CD45	Alexa Fluor 700	BioLegend	103128	1:100
CD11b	PerCP-Cy5.5	eBioscience	45-0112-82	1:100
Ly-6G (GR-1)	FITC	FISHER SCIENTIFIC SAS	11-5931-82	1:100
Ly-6C	PE-CF594	BD Biosciences	562728	1:100
F4/80	APC eFluor 780	Invitrogen	47-4801-80	1:100
CD3ε	PerCP-Cy5.5	BioLegend	100328	1:50
CD4	APC-H7	BD Pharmingen™	560181	1:100
CD8a	Alexa Fluor 700	eBioscience	56-0081-80	1:100

Supplementary Table 5. List of the antibodies used for Th cells isolation and differentiation.

Antigen	Conjugation	Company	Cat. number	Dilution
CD16/CD32	purified	BioLegend	101302	1:100
CD4	AlexaFluor 700	BioLegend	100536	1:100
CD8a	PerCP-Cy5.5	BioLegend	100734	1:100
CD44	PE-Cy7	eBioscience	25-0441	1:500
CD25	PE	BioLegend	102008	1:300
Nk1.1	PE	BioLegend	108708	1:300
TCR gd	PE	BD Biosciences	553178	1:100
CD3ε	Ultra-leaf	BioLegend	100359	1:50
CD28	Ultra-leaf	BioLegend	102121	1:100
IFN-g	Ultra-leaf	BioLegend	505847	1:100
IL-4	Ultra-leaf	BioLegend	504135	1:100
IL-17	APC	eBioscience	17-7177	1:100
IFN-g	PE-Dazzle 594	BioLegend	505846	1:100
