# Supplementary material for: LSD1 inhibition circumvents glucocorticoid-induced muscle wasting of male mice
Source: Nat Commun. 2024 Apr 26;15:3563. doi: 10.1038/s41467-024-47846-9 (PMC11053113; doi:10.1038/s41467-024-47846-9)
Supplement: Supplementary file 3 — Reporting Summary [file 41467_2024_47846_MOESM3_ESM.pdf]

nature portfolio

Corresponding author(s): Delphine Duteil

Last updated by author(s): 25/03/2024

Reporting Summary

Nature Portfolio wishes to improve the reproducibility of the work that we publish. This form provides structure for consistency and transparency in reporting. For further information on Nature Portfolio policies, see our Editorial Policies and the Editorial Policy Checklist.

Statistics

For all statistical analyses, confirm that the following items are present in the figure legend, table legend, main text, or Methods section.

n/a	Confirmed	
☐	☒	The exact sample size (*n*) for each experimental group/condition, given as a discrete number and unit of measurement
☐	☒	A statement on whether measurements were taken from distinct samples or whether the same sample was measured repeatedly
☐	☒	The statistical test(s) used AND whether they are one- or two-sided<br>*Only common tests should be described solely by name; describe more complex techniques in the Methods section.*
☒	☐	A description of all covariates tested
☐	☒	A description of any assumptions or corrections, such as tests of normality and adjustment for multiple comparisons
☒	☐	A full description of the statistical parameters including central tendency (e.g. means) or other basic estimates (e.g. regression coefficient) AND variation (e.g. standard deviation) or associated estimates of uncertainty (e.g. confidence intervals)
☒	☐	For null hypothesis testing, the test statistic (e.g. *F*, *t*, *r*) with confidence intervals, effect sizes, degrees of freedom and *P* value noted<br>*Give P values as exact values whenever suitable.*
☒	☐	For Bayesian analysis, information on the choice of priors and Markov chain Monte Carlo settings
☐	☒	For hierarchical and complex designs, identification of the appropriate level for tests and full reporting of outcomes
☒	☐	Estimates of effect sizes (e.g. Cohen's *d*, Pearson's *r*), indicating how they were calculated

Our web collection on statistics for biologists contains articles on many of the points above.

Software and code

Policy information about availability of computer code

Data collection

All softwares are commercially or freely available. Images were captured using a Leica DM 4000 B or Leica TCS SP8 laser scanning microscope. Flow cytometry analysis was performed on a BD LSR II flow cytometer or FACS ARIA Fusion (BD Biosciences). For H&E staining and ultrastructural analyses, slides were acquired using a NanoZoomer S210 scanner (Hamamatsu) and a Mega View III camera (Soft Imaging System), respectively. Quantitative PCR was performed with a Lightcycler 480 II (Roche). AI 600 imager (GE Healthcare Life Science) was used to collect Western Blot images. For RNA-seq and ChIP-seq, RNA integrity and DNA quality were measured and the library was quantified using Agilent 2100 Bioanalyzer (Agilent, Santa Clara, CA). RNA-seq sequencing was performed on an Illumina HiSeq 2000 system and ChIP-seq on an Illumina HiSeq 4000.

Data analysis

All softwares are commercially or freely available. Flow cytometry analysis was performed on FlowJo 10.8.1. The lightCycler480 software, microsoft Excel and GraphPad Prism were used for analysis of qPCR data. Image J-based Fiji was used to quantify Western blotting. Qupath Imaged was used to analyze Immunohistochemistry. GraphPad Prism was used for analysis data and carry out statistical analysis. For ChIP-seq analysis, reads were mapped onto the mm10 reference genome using Bowtie 1.1.2. Uniquely mapped reads were retained for further analysis. Reads overlapping with ENCODE hg38 blacklisted region V2 were removed using Bedtools73. Bigwig files were generated using Homer74 software makeUCSCfile script with default parameters and scaled to 1e7 reads. MACS2 (2.2.7.1) algorithm (https://github.com/taoliu/MACS/) was used for the peak calling and the appropriate input DNA from each sample was used as control. All peaks with an FDR greater than 0.01 were excluded from further analysis. The genome-wide intensity profiles were visualized using the IGV genome browser (http://software.broadinstitute.org/software/igv/). HOMER was used to annotate peaks and for motif searches. De novo identified motifs were referred to as follow: R = purine (G or A); Y = pyrimidine (T or C). Genomic features (promoter/TSS, 5' UTR, exon, intron, 3' UTR, TTS and intergenic regions) were defined and calculated using Refseq and HOMER according to the distance to the nearest TSS. Clustering analyses were done with the seqMINER software, and clustering normalization was done with the K-Means linear option. Venn diagrams were generated with Venny (https://bioinfogp.cnb.csic.es/tools/venny/). Pathway analysis was performed with WebGestalt using the Over-

Representation Analysis (ORA) method. Parameters were set as default, with the exception of the following: Bowtie (-m 1 -- strata -- best - y -S - l 40), MACS2 [callpeak -- gsize 1.87e9 -- nomodel -- extsize 150 -- broad --keep-dup auto], seqMINER (input bed files normalized to 20 million reads per sample). For RNA-seq analysis, image analysis and base calling were performed using RTA 2.7.7 and bcl2fastq 2.17.1.14. Adapter dimer reads were removed using DimerRemover (-a AGATCGGAAGAGCACACGTCTGAACTCCAGTCAC). FastQC 0.11.2 (http://www.bioinformatics.babraham.ac.uk/projects/fastqc/) was used to evaluate the quality of sequencing. Reads were mapped to the mouse mm10 genome (NCBI Build 38) using htseq-count (Version 0.9.1). Only uniquely aligned reads were retained for further analyses. Quantification of gene expression was performed using HOMER (http://homer.ucsd.edu/homer/ngs/analyzeRNA.html). For comparison among datasets, transcripts with more than 50 raw reads were considered. Differentially expressed genes (DEGs) were identified using the Bioconductor libraries DESeq2. A p-value < 0.05 and a fold change excluding values between 0.77 and 1.3 were used as a threshold for DEGs, and further submitted for pathway analysis in WebGestalt using the Over-Representation Analysis (ORA) method and a p-value < 0.05. Heatmaps were generated by centering and normalizing expression values with Cluster 3.0 and importing them to MORPHEUS (https://software.broadinstitute.org/morpheus/). Genes were clustered according to the hierarchical method (HCL clustering) using gene tree, the Pearson correlation and average linkage.

For manuscripts utilizing custom algorithms or software that are central to the research but not yet described in published literature, software must be made available to editors and reviewers. We strongly encourage code deposition in a community repository (e.g. GitHub). See the Nature Portfolio guidelines for submitting code & software for further information.

Data

Policy information about availability of data

All manuscripts must include a data availability statement. This statement should provide the following information, where applicable:

- Accession codes, unique identifiers, or web links for publicly available datasets
- A description of any restrictions on data availability
- For clinical datasets or third party data, please ensure that the statement adheres to our policy

The raw and processed high-throughput sequencing datasets including RNA-seq and ChIP-seq data generated in this study have been deposited to the Gene Expression Omnibus (GEO) database under the accession number GSE230547. All remaining data is available in the Article, Supplementary and Source Data files. Source data are provided with this paper.

Research involving human participants, their data, or biological material

Policy information about studies with human participants or human data. See also policy information about sex, gender (identity/presentation), and sexual orientation and race, ethnicity and racism.

Reporting on sex and gender	Not applicable
Reporting on race, ethnicity, or other socially relevant groupings	Not applicable
Population characteristics	Not applicable
Recruitment	Not applicable
Ethics oversight	Not applicable

Note that full information on the approval of the study protocol must also be provided in the manuscript.

Field-specific reporting

Please select the one below that is the best fit for your research. If you are not sure, read the appropriate sections before making your selection.

☒ Life sciences ☐ Behavioural & social sciences ☐ Ecological, evolutionary & environmental sciences

For a reference copy of the document with all sections, see nature.com/documents/nr-reporting-summary-flat.pdf

Life sciences study design

All studies must disclose on these points even when the disclosure is negative.

Sample size	Sample size was chosen based on experience with the used experimental models in the field of cell biology and animal experiments. Detailed description of the statistical methods used for the analyse, appears in the paper. For molecular experiments, the results were collected from at least three biological replicates, as described in figure legends, to ensure consistent phenotypes and to perform statistical analyses.
Data exclusions	No data were excluded throughout the studies
Replication	The replication numbers were described in the corresponding figure legends.
Randomization	For in vivo experiments, age matched mice were randomized into all experimental groups. All the cell experiments in vitro were performed by

Randomization	plating the cells in independent dishes or plates, and randomly assigned to experimental or control groups and no bias was introduced.
Blinding	The investigators were blind to the genotypes of mice and relied solely on identification numbers throughout the study.

Reporting for specific materials, systems and methods

We require information from authors about some types of materials, experimental systems and methods used in many studies. Here, indicate whether each material, system or method listed is relevant to your study. If you are not sure if a list item applies to your research, read the appropriate section before selecting a response.

Materials & experimental systems

n/a	Involved in the study	
☐	☒	Antibodies
☐	☒	Eukaryotic cell lines
☒	☐	Palaeontology and archaeology
☐	☒	Animals and other organisms
☒	☐	Clinical data
☒	☐	Dual use research of concern
☒	☐	Plants

Methods

n/a	Involved in the study	
☐	☒	ChIP-seq
☐	☒	Flow cytometry
☒	☐	MRI-based neuroimaging

Antibodies

Antibodies used

The following antibodies for western blot analysis were diluted as followed:
Rabbit anti-LSD1 (N-terminal, R. Schüle, #3544, 1:1000)
Rabbit anti-GR (C-terminal, IGBMC, #3249, 1:500)
NRF1 (Clone 2F9, Abcam, ab55744, 1:1000)
phospho-mTOR (Ser2448, Clone 49F9, Cell Signaling, #2976S, 1/1000)
mTOR (Clone 7C10, Cell Signaling, #2983S, 1/500)
phospho-4E-BP1 (Thr37/46, Clone 236B4, Cell Signaling, #2855, 1/1500)
4E-BP1 (Clone 53H11, Cell Signaling, # 9644, 1/1500)
phospho-FOXO3a (Ser318/321, Cell Signaling, #9465, 1/1000)
FOXO3a (Clone 75D8, Cell Signaling, #2497, 1/1000)
phospho-Akt (Thr308, Abcam, ab38449, 1/1000)
Akt1 (C67E7, Cell Signaling, #4691, 1/500)
phospho-GSK3B (Ser9, D85E12, Cell Signaling, #5558, 1/1500)
GSK3B (Clone 7, BD Transduction Laboratories, #610201, 1/1000)
LC3B (Clone GT1187, Genetex, 1/1000)
P62 (Clone 2C11, abcam, ab56416, 1/5000)
β-ACTIN (sc-47778, Santa Cruz, 1:5000)
α-TUBULIN (IGBMC, 1Tub2A2, 1:5000)
GAPDH (Clone 14C10, Cell Signaling, #2118, 1:5000)
Secondary antibodies conjugated to horseradish peroxidase (Jackson ImmunoResearch, 1:10000)
The following antibodies were used to perform immunofluorescence:
For muscle tissues:
Rabbit-anti-LSD1 (N-terminal, R. Schüle, #3544, 1:500)
Mouse-anti-GR (Santa Cruz, sc393232, 1:500)
Rabbit IgGs (Santa Cruz, sc2357, 1:1000)
Mouse IgGs (Santa Cruz, sc 2025, 1:1000)
For LHCN-M2 cells:
Rabbit-anti-LSD1 (N-terminal, R. Schüle, #3544, 1:500)
Mouse-anti-GR (Invitrogen, MA1-510, 1:500)
The following antibodies were used to perform ChIP:
Rabbit anti-GR (C-terminal, IGBMC, #3249, 5 µg)
Rabbit anti-LSD1 (C-terminal, R. Schüle, #20752, 5 µg)
Rabbit anti-NRF1 (Abcam, ab175932, 5 µg)
H3K9me1 (Active Motif, #39249, 5 µg)
H3K9me2 (Active Motif, #39239, 5 µg)
Rabbit IgG (Santa Cruz, sc2357, 5 µg)
The following antibodies were used to perform Flow cytometry:
For spleen:
CD45 (Alexa Fluor 700, Clone 30-F11, BioLegend, cat. 103128, 1:100)
CD11b (PerCP-Cy5.5, Clone M1/70, eBioscience, cat. 45-0112-82, 1:100)
Ly-6G (GR-1) (FITC, Clone RB6-8C5, FISHER SCIENTIFIC SAS, cat. 11-5931-8,2 1:100)
Ly-6C (PE-CF594, BD Biosciences, cat. 562728, 1:100)
F4/80 (APC eFluor 780, Clone BM8, Invitrogen, cat. 47-4801-80, 1:100)
EPCAM (PE-Cy7, Clone 8.8, BioLegend, cat. 118216, 1:100)
CD3ε (PerCP-Cy5.5, Clone 145-2C11, BioLegend, cat. 100328, 1:50)
CD4 (APC-H7, BD Biosciences, cat. 560181, 1:100)
CD8a (Alexa Fluor 700, Clone 53-6.7, eBioscience, cat. 56-0081-80, 1:100)

CD49b (FITC, Clone DX5, eBioscience, cat. 11-5971-82, 1:100)
NK1.1 (PerCP-Cy7, Clone PK136, eBioscience, cat. 25-5941-82, 1:1000)
CD25 (Alexa Fluor 780, Clone 61, BioLegend, cat. 102038, 1:100)
For peripheral and mesenteric lymph nodes cells:
CD16/CD32 (purified, Clone 93, BioLegend, cat. 101302, 1:100)
CD4 (AlexaFluor 700, Clone RM4-5, BioLegend, cat. 100536, 1:100)
CD8a (PerCP-Cy5.5, Clone 53-6.7, BioLegend, cat. 100734, 1:100)
CD44 (PE-Cy7, Clone IM7, eBioscience, cat. 25-0441, 1:500)
CD25 (PE, Clone 67, BioLegend, cat. 102008, 1:300)
Nk1.1 (PE, Clone PK136, BioLegend, cat. 108708, 1:300)
TCR gd (PE, Clone GL3, BD Biosciences, cat. 553178, 1:100)
CD3ε (Ultra-leaf, Clone 145-2C11, BioLegend, cat. 100359, 1:50)
CD28 (Ultra-leaf, Clone 37.51, BioLegend, cat. 102121, 1:100)
IFN-g (Ultra-leaf, Clone XMG1.2, BioLegend, cat. 505847, 1:100)
IL-4 (Ultra-leaf, Clone 11B11, BioLegend, cat. 504135, 1:100)
IL-17 (APC, Clone eBio17B7, eBioscience, cat. 17-7177, 1:100)
IFN-g (PE-Dazzle 594, Clone XMG1.2, BioLegend, cat. 505846, 1:100)

Validation

All antibodies used in this study are commercially available, and were otherwise validated by the manufacturer, by previous studies from other laboratories or by previous studies from our laboratory, as cited in the text and methods.
Antibody
The primary antibodies for western blot analysis were diluted as followed:
LSD1 (N-terminal, R. Schüle, #3544, 1:1000)
https://www.sciencedirect.com/science/article/pii/S221112471631292X?via%3Dihub
Rabbit-anti GR (C-terminal, IGBMC, #3249, 1:500)
https://academic.oup.com/nar/article/49/8/4472/6219119
Mouse-anti-GR (Clone G-5, Santa Cruz, sc393232, 1:500)
https://www.scbt.com/zh/p/gr-antibody-g-5
Mouse-anti-GR (BuGR2, Invitrogen, MA1-510, 1:500)
https://www.thermofisher.com/antibody/product/Glucocorticoid-Receptor-Antibody-clone-BuGR2-Monoclonal/MA1-510
NRF1 (Clone 2F9, Abcam, ab55744, 1:1000)
https://www.abcam.com/products/primary-antibodies/nrf1-antibody-2f9-ab55744.html
phospho-mTOR (Ser2448, Clone 49F9, Cell Signaling, #2976S, 1/1000)
https://www.cellsignal.com/products/primary-antibodies/phospho-mtor-ser2448-49f9-rabbit-mab/2976
mTOR (Clone 7C10, Cell Signaling, #2983S, 1/500)
https://www.cellsignal.com/products/primary-antibodies/mtor-7c10-rabbit-mab/2983
phospho-4E-BP1 (Thr37/46, Clone 236B4, Cell Signaling, #2855, 1/1500)
https://www.cellsignal.com/products/primary-antibodies/phospho-4e-bp1-thr37-46-236b4-rabbit-mab/2855
4E-BP1 (Clone 53H11, Cell Signaling, # 9644, 1/1500)
https://www.cellsignal.com/products/primary-antibodies/4e-bp1-53h11-rabbit-mab/9644
phospho-FOXO3a (Ser318/321, Cell Signaling, #9465, 1/1000)
https://www.cellsignal.cn/products/primary-antibodies/phospho-foxo3a-ser318-321-antibody/9465
FOXO3a (Clone 75D8, Cell Signaling, #2497, 1/1000)
https://www.cellsignal.com/products/primary-antibodies/foxo3a-75d8-rabbit-mab/2497
phospho-Akt (Thr308, Abcam, ab38449, 1/1000)
https://www.abcam.com/products/primary-antibodies/akt-phospho-t308-antibody-ab38449.html
Akt1 (C67E7, Cell Signaling, #4691, 1/500)
https://www.cellsignal.com/products/primary-antibodies/akt-pan-c67e7-rabbit-mab/4691
phospho-GSK3B (Ser9, D85E12, Cell Signaling, #5558, 1/1500)
https://www.cellsignal.com/products/primary-antibodies/phospho-gsk-3b-ser9-d85e12-xp-rabbit-mab/5558
GSK3B (Clone 7, BD Transduction Laboratories, #610201, 1/1000)
https://www.bdbiosciences.com/en-us/products/reagents/microscopy-imaging-reagents/immunofluorescence-reagents/purified-mouse-anti-gsk-3.610202
LC3B (Clone GT1187, Genetex, 1/1000)
https://www.genetex.com/Product/Detail/LC3B-antibody-GT1187/GTX00949
P62 (Clone 2C11, abcam, ab56416, 1/5000)
https://www.abcam.com/products/primary-antibodies/sqstm1--p62-antibody-2c11-bsa-and-azide-free-ab56416.html
β-ACTIN (sc-47778, Santa Cruz, 1:5000)
https://www.scbt.com/p/beta-actin-antibody-c4?requestFrom=search
α-TUBULIN (IGBMC, 1Tub2A2, 1:5000)
https://academic.oup.com/nar/article/49/8/4472/6219119
GAPDH (Clone 14C10, Cell Signaling, #2118, 1:5000)
https://www.cellsignal.com/products/primary-antibodies/gapdh-14c10-rabbit-mab/2118
Rabbit IgGs (Santa Cruz, sc2357, 1:1000)
https://www.scbt.com/zh/p/mouse-anti-rabbit-igg-hrp
Mouse IgGs (Santa Cruz, sc2025, 1:1000)
https://www.scbt.com/zh/p/normal-mouse-igg
Rabbit anti-LSD1 (C-terminal, R. Schüle, #20752, 5 μg)
https://www.sciencedirect.com/science/article/pii/S221112471631292X?via%3Dihub
Rabbit anti-NRF1 (Clone EPR5554N, Abcam, ab175932, 5 μg)
https://www.abcam.com/en-nl/products/primary-antibodies/nrf1-antibody-epr5554n-chip-grade-ab175932
H3K9me1 (Active Motif, #39249, 5 μg)
https://www.activemotif.com/catalog/details/39249/histone-h3-monomethyl-lys9-antibody-pab-1
H3K9me2 (Active Motif, #39239, 5 μg)
https://www.activemotif.com/catalog/details/39239/histone-h3-dimethyl-lys9-antibody-pab
CD45 (Alexa Fluor 700, Clone 30-F11, BioLegend, cat. 103128, 1:100)

https://www.biolegend.com/en-us/products/alexa-fluor-700-anti-mouse-cd45-antibody-3407
CD11b (PerCP-Cy5.5, Clone M1/70, eBioscience, cat. 45-0112-82, 1:100)
https://www.thermofisher.com/antibody/product/CD11b-Antibody-clone-M1-70-Monoclonal/45-0112-82
Ly-6G (GR-1) (FITC, Clone RB6-8C5, FISHER SCIENTIFIC SAS, cat. 11-5931-8,2 1:100)
https://www.thermofisher.com/antibody/product/Ly-6G-Ly-6C-Antibody-clone-RB6-8C5-Monoclonal/11-5931-82
Ly-6C (PE-CF594, BD Biosciences, cat. 562728, 1:100)
https://www.bdbiosciences.com/en-fr/products/reagents/flow-cytometry-reagents/research-reagents/single-color-antibodies-ruo/pe-cf594-rat-anti-mouse-ly-6c.562728
F4/80 (APC eFluor 780, Clone BM8, Invitrogen, cat. 47-4801-80, 1:100)
https://www.thermofisher.com/antibody/product/F4-80-Antibody-clone-BM8-Monoclonal/47-4801-80
EPCAM (PE-Cy7, Clone 8.8, BioLegend, cat. 118216, 1:100)
https://www.biolegend.com/en-us/products/pe-cyanine7-anti-mouse-cd326-ep-cam-antibody-5303
CD3ε (PerCP-Cy5.5, Clone 145-2C11, BioLegend, cat. 100328, 1:50)
https://www.biolegend.com/en-us/products/percp-cyanine5-5-anti-mouse-cd3epsilon-antibody-4191
CD4 (APC-H7, BD Biosciences, cat. 560181, 1:100)
https://www.bdbiosciences.com/en-ca/products/reagents/flow-cytometry-reagents/research-reagents/single-color-antibodies-ruo/apc-h7-rat-anti-mouse-cd4.560181
CD8a (Alexa Fluor 700, Clone 53-6.7, eBioscience, cat. 56-0081-80, 1:100)
https://www.thermofisher.com/antibody/product/CD8a-Antibody-clone-53-6-7-Monoclonal/56-0081-80
CD49b (FITC, Clone DX5, eBioscience, cat. 11-5971-82, 1:100)
https://www.thermofisher.com/antibody/product/CD49b-Integrin-alpha-2-Antibody-clone-DX5-Monoclonal/11-5971-82
NK1.1 (PerCP-Cy7, Clone PK136, eBioscience, cat. 25-5941-82, 1:1000)
https://www.thermofisher.com/antibody/product/NK1-1-Antibody-clone-PK136-Monoclonal/25-5941-82
CD25 (Alexa Fluor 780, Clone 61, BioLegend, cat. 102038, 1:100)
https://www.biolegend.com/en-us/products/brilliant-violet-650-anti-mouse-cd25-antibody-7640
For peripheral and mesenteric lymph nodes cells:
CD16/CD32 (purified, Clone 93, BioLegend, cat. 101302, 1:100)
https://www.biolegend.com/en-us/search-results/purified-anti-mouse-cd16-32-antibody-190?gclid=CjwKCAiAjrarBhAWEiwA2qWdCEt7IFidszzxZVoVGEbdvYa3eoh8DyKuSIYJit7xY3-pVUJ407d-fRoCNY4QAvD_BwE
CD4 (AlexaFluor 700, Clone RM4-5, BioLegend, cat. 100536, 1:100)
https://www.biolegend.com/en-us/products/alexa-fluor-700-anti-mouse-cd4-antibody-3386
CD8a (PerCP-Cy5.5, Clone 53-6.7, BioLegend, cat. 100734, 1:100)
https://www.biolegend.com/en-us/products/percp-cyanine5-5-anti-mouse-cd8a-antibody-4255
CD44 (PE-Cy7, Clone IM7, eBioscience, cat. 25-0441, 1:500)
https://www.thermofisher.com/antibody/product/CD44-Antibody-clone-IM7-Monoclonal/25-0441-82
CD25 (PE, Clone 67, BioLegend, cat. 102008, 1:300)
https://www.biolegend.com/en-us/products/pe-anti-mouse-cd25-antibody-424
Nk1.1 (PE, Clone PK136, BioLegend, cat. 108708, 1:300)
https://www.biolegend.com/en-us/products/pe-anti-mouse-nk-1-1-antibody-431
TCR gd (PE, Clone GL3, BD Biosciences, cat. 553178, 1:100)
https://www.bdbiosciences.com/en-fr/products/reagents/flow-cytometry-reagents/research-reagents/single-color-antibodies-ruo/pe-hamster-anti-mouse-t-cell-receptor.553178
CD3ε (Ultra-leaf, Clone 145-2C11, BioLegend, cat. 100359, 1:50)
https://www.biolegend.com/en-us/products/ultra-leaf-purified-anti-mouse-cd3epsilon-antibody-7722
CD28 (Ultra-leaf, Clone 37.51, BioLegend, cat. 102121, 1:100)
https://www.biolegend.com/en-us/products/ultra-leaf-purified-anti-mouse-cd28-antibody-7733
IFN-g (Ultra-leaf, Clone XMG1.2, BioLegend, cat. 505847, 1:100)
https://www.biolegend.com/en-us/products/ultra-leaf-purified-anti-mouse-ifn-gamma-antibody-7752
IL-4 (Ultra-leaf, Clone 11B11, BioLegend, cat. 504135, 1:100)
https://www.biolegend.com/en-us/products/ultra-leaf-purified-anti-mouse-il-4-antibody-7750
IL-17 (APC, Clone eBio17B7, eBioscience, cat. 17-7177, 1:100)
https://www.thermofisher.com/antibody/product/IL-17A-Antibody-clone-eBio17B7-Monoclonal/17-7177-81
IFN-g (PE-Dazzle 594, Clone XMG1.2, BioLegend, cat. 505846, 1:100)
https://www.biolegend.com/en-us/products/pe-dazzle-594-anti-mouse-ifn-gamma-antibody-9986

Eukaryotic cell lines

Policy information about cell lines and Sex and Gender in Research

Cell line source(s)	C2C12 myoblasts, obtained from ATCC (CRL-1772)<br>LHCN-M2 were kindly gifted by Dr. Jocelyn LAPORTE (IGBMC, Strasbourg University, France).
Authentication	C2C12 and LHCN-M2 were not authenticated.
Mycoplasma contamination	All the cells used in this study were tested Mycoplasma-negative.
Commonly misidentified lines<br>(See ICLAC register)	No commonly misidentified cell lines were used in this study.

Animals and other research organisms

Policy information about studies involving animals; ARRIVE guidelines recommended for reporting animal research, and Sex and Gender in Research

Laboratory animals	Mice were housed under specific pathogen-free conditions with controlled temperature (19-23°C) and humidity (40-60%) on a 12-h light/dark cycle with unrestricted access to standard laboratory rodent chow (2800 kcal/kg, Usine d'Alimentation Rationelle, Villemoisson-sur-Orge, France) and water were provided ad libitum. Breeding and maintenance of mice were performed according to institutional guidelines. The following mouse lines were used: HSA-Cre/LSD1L2/L2 (also named LSD1skm-/-), HSA-CreERT2/LSD1L2/L2 (also named LSD1(i)skm-/-), and HSA-CreERT2/LSD1L2/L2 (also named GR(i)skm-/-) mice. LSD1L2/L2, and GRL2/L2 floxed mice were used as control. All mice used were on C57BL/6J background and tissues were harvested from 9 or 12 week old male mice.
Wild animals	C56/BL6 wild-type animals were in the study.
Reporting on sex	Studies were performed on male mice.
Field-collected samples	No field-collected samples were used in this study.
Ethics oversight	All experiments were done in an accredited animal house, in compliance with French and EU regulations on the use of laboratory animals for research. Intended manipulations were submitted to the Ethical committee (Com'Eth, Strasbourg, France) for approval and to the French Research Ministry (MESR) for ethical evaluation and authorization according to the 2010/63/EU directive under the APAFIS numbers (2015-26, 37660, 39468 and 45167).

Note that full information on the approval of the study protocol must also be provided in the manuscript.

Plants

Seed stocks	Not applicable
Novel plant genotypes	Not applicable
Authentication	Not applicable

ChIP-seq

Data deposition

☒ Confirm that both raw and final processed data have been deposited in a public database such as GEO.

☒ Confirm that you have deposited or provided access to graph files (e.g. BED files) for the called peaks.

Data access links<br>*May remain private before publication.*	ChIP-seq data reported in this study are available at GEO database at GSE230547.
Files in database submission	For ChIP-seq:<br>- fastq files:<br>MLVQ1.R1.fastq.gz<br>MLVQ2.R1.fastq.gz<br>MLVQ4.R1.fastq.gz<br>MLVQ5.R1.fastq.gz<br>MLVQ6.R1.fastq.gz<br>MLVQ15.R1.fastq.gz<br>MLVQ16.R1.fastq.gz<br>MLVQ21.R1.fastq.gz<br>MLVQ27.R1.fastq.gz<br>MLVQ28.R1.fastq.gz<br>MLVQ91.R1.fastq.gz<br>MLVQ93.R1.fastq.gz<br>- big wig files:<br>GR_limb_MLVQ1.bigwig<br>Lsd1_20752_limb_MLVQ2.bigwig<br>H3K9me1_limb_MLQV4.bigwig<br>H3K9me2_limb_MLQV5.bigwig<br>Input_limb_MLQV6.bigwig<br>24h_Lsd1_20752_limb_MLVQ15.bigwig

24h_GR_limb_MLVQ16.bigwig
Input_24h_limb_MLVQ21.bigwig
24h_H3K9me1_limb_MLVQ27.bigwig
24h_H3K9me2_limb_MLVQ28.bigwig
NRF1_MLVQ91.ucsc.bw
Input_NRF1_MLVQ93.ucsc.bw
- bed files:
GR.bed.gz
Lsd1.bed.gz
H3K9me1.bed.gz
H3K9me2.bed.gz
24h_Lsd1_20752.bed.gz
24h_GR.bed.gz
24h_H3K9me1.bed.gz
24h_H3K9me2.bed.gz
Narrow_Peaks.bed.gz (for NRF1)
For RNA-seq:
- fastq files:
MLVQ45.R1.fastq.gz
MLVQ46.R1.fastq.gz
MLVQ47.R1.fastq.gz
MLVQ48.R1.fastq.gz
MLVQ49.R1.fastq.gz
MLVQ50.R1.fastq.gz
MLVQ51.R1.fastq.gz
MLVQ52.R1.fastq.gz
-processed file:
S22186_read_counts.txt

Genome browser session (e.g. UCSC): IGV_2.15.1

Methodology

Replicates: Experiments are repeated at least 3 times, and the number of mice per condition is of at least 3.

Sequencing depth: Illumina Hiseq 4000 as single-end 50 bp reads

Antibodies: Rabbit-anti GR (C-terminal, IGBMC, #3249); Rabbit anti-LSD1 (C-terminal, R. Schüle, #20752); Rabbit anti-NRF1 (Clone EPR5554N, Abcam, ab175932); H3K9me1 (Active Motif, #39249); H3K9me2 (Active Motif, #39239)

Peak calling parameters: MACS2 (2.2.7.1) algorithm (https://github.com/taoliu/MACS/) was used for the peak calling and the appropriate input DNA from each sample was used as control. All peaks with an FDR greater than 0.01 were excluded from further analysis.

Data quality: For LSD1 ChIP-seq, 16616 peaks were obtained with a FDR < 0.01 and 5<m-fold<50. Using same parameters, 14108 peaks were obtained for GR (in agreement with doi: 10.1093/nar/gkab226) and 3130 for NRF1. Upon starvation, 37783 peaks were obtained for GR and 692 for LSD1.

Software: Reads were mapped onto the mm10 reference genome using Bowtie 1.1.2. Uniquely mapped reads were retained for further analysis. Reads overlapping with ENCODE hg38 blacklisted region V2 were removed using Bedtools73. Bigwig files were generated using Homer74 software makeUCSCfile script with default parameters and scaled to 1e7 reads. MACS2 (2.2.7.1) algorithm (https://github.com/taoliu/MACS/) was used for the peak calling and the appropriate input DNA from each sample was used as control. All peaks with an FDR greater than 0.01 were excluded from further analysis. The genome-wide intensity profiles were visualized using the IGV genome browser (http://software.broadinstitute.org/software/igv/). HOMER was used to annotate peaks and for motif searches. De novo identified motifs were referred to as follow: R = purine (G or A); Y = pyrimidine (T or C). Genomic features (promoter/TSS, 5' UTR, exon, intron, 3' UTR, TTS and intergenic regions) were defined and calculated using Refseq and HOMER according to the distance to the nearest TSS. Clustering analyses were done with the seqMINER software, and clustering normalization was done with the K-Means linear option. Venn diagrams were generated with Venny (https://bioinfogp.cnb.csic.es/tools/venny/). Pathway analysis was performed with WebGestalt using the Over-Representation Analysis (ORA) method.

Flow Cytometry

Plots

Confirm that:

☒ The axis labels state the marker and fluorochrome used (e.g. CD4-FITC).

☒ The axis scales are clearly visible. Include numbers along axes only for bottom left plot of group (a 'group' is an analysis of identical markers).

☒ All plots are contour plots with outliers or pseudocolor plots.

☒ A numerical value for number of cells or percentage (with statistics) is provided.

Methodology

Sample preparation

For spleen, tissues were transferred into a sterile 35 mm culture dish containing 5 mL of phosphate-buffered saline (PBS) with 1 mM EDTA, and crushed with the flat end of a sterile 3 cc syringe plunger. A 70 µm cell strainer was used to eliminate clumps, and the suspension was washed in PBS.
For peripheral and mesenteric lymph nodes cells, cells of C57BL/6 mice were stained anti-CD16/CD32 blocking antibodies, anti-CD4, anti-CD8, anti-CD44, anti-CD25, anti-NK1.1, and anti-TCRgd antibodies in PBS 10% heat-inactivated FCS for 15 min on ice. Naive CD4 T cells (CD4+, CD8-, CD44lo, TCRgd-, NK1.1-) were sorted using a FACS ARIA Fusion (BD Biosciences) with a purity > 98%. Naïve CD4 T cells (4x104/well) were then activated with anti-CD3 (clone 2C11) and anti-CD28 (clone 37.51) antibodies, both pre-coated overnight in PBS at 4°C on a nunc-immuno 96 well plate, with (Th17 conditions) or without (Th0 conditions) IL-6 (10 ng/ml) and TGFb1 (0.125 ng/ml) in the presence of neutralizing anti-IFNg and anti-IL-4 Abs (10 µg/ml each) in Iscove's Modified Dulbecco's Medium (IMDM) containing 10 % inactivated FCS, Glutamax, 10 mM Hepes, sodium pyruvate and B-mercaptoethanol (200 µl/well). After 3 days of culture in the presence of vehicle, DEX (100 nM) and/or CC-90011 (100 nM), cells were stimulated with phorbol-12-myristate-13-acetate (PMA) plus ionomycin (0.5 µg/ml each) and GolgiPlug (1/1000) for 2h, stained with the BD HorizonTM fixable viability stain 780 Zombie, then with anti-CD4 antibody, fixed and permeabilized using the Intracellular Fix & permeabilization set (eBioscience) and stained with anti-IL-17 and anti-IFNg antibodies.

Instrument

BD FACS Celesta™ Cell Analyzer (BD Bioscience) for spleen and a FACS ARIA Fusion (BD Biosciences) for peripheral and mesenteric lymph nodes cells.

Software

FlowJo 10.8.1 Software

Cell population abundance

n/a

Gating strategy

Cells were selected according to the FCS-A and SSC-A (>20) to exclude debris.
Duplets were removed with the FCS-A and FSC-H gating.
An unstained negative sample was first used to set up the gatings for the various antibodies and dyes.
Dead cells were excluded with DAPI <1e3.
Immune cells were selected as CD45 > 1e2.

☒ Tick this box to confirm that a figure exemplifying the gating strategy is provided in the Supplementary Information.
